# The Coming of Age of the Fund.—A Distribution of £200,000

**Published:** 1918-12-21

**Authors:** 


### KING EDWARD'S HOSPITAL FUND FOR LONDON.

### The Coming of Age of the Fund.—A Distribution of £200,000.

A meeting of the Governors and General Council of King Edward's Hospital Fund for London, for the purpose of awarding grants to the hospitals, convalescent homes, and consumption sanatoria for the present year, was held on Tuesday, December 17, at St. James's Palace, the **Marquis of Cambridge** being in the chair.

Lord Revelstoke (the Honorary Treasurer) said that he had pleasure in stating that the total receipts on General Fund to December 7, after deducting expenses, amounted to £174,531. The total receipts for the year, exclusive of the contribution from the League of Mercy, to which reference would duly be made later on by Sir William Collins, were, however, estimated to amount to £178,271. While these were the receipts for the year, he was able to report that the Fund had with its bankers, or at short call, sufficient cash to meet the whole of the sum which it had been decided to distribute for the year 1918, amounting in all to £200,000.

The League of Mercy's Twenty Years' Work.

Sir William Collins, in making the annual statement on behalf of the League of Mercy, reminded the Council that in the last complete pre-war year the contribution from the League to the Fund was £14,000; in 1914 and 1915 the same amount; in 1916 and 1917 £15,000. He was glad to be able to say that the League would be able this year to present £16,000. That would make a total contribution, during the five years of war, of £74,000, as against £14,000 in the last pre-war year, and an average of £13,500 in all the pre-war years. The League had been in existence for just twenty years; during that period it would have contributed, with the amount announced this day, £276,000 to King Edward's Fund, and to the extra-Metropolitan hospitals in the area of its collection outside the area of distribution of the King's Fund £29,280, making for the twenty years a total contribution from the League of Mercy to the voluntary hospitals in and around London of £305,280.

### His Majesty on Progress and Prospects.

The **Marquis of Cambridge**, in moving the adoption of the Reports and Awards, said :—

My Lords and Gentlemen,—Before moving the adoption of the Reports I have the honour to read the following message which His Majesty the King, Patron of the Fund, has been graciously pleased to send through Lord Stamfordham :—

"Buckingham Palace,
"December 16, 1918.

"Dear Sir,—I am commanded by the King, on this the coming of age of the Fund which bears his father's name, and was founded by his late Majesty in 1897, to convey to the Governors and General Council his hearty congratulations on the success which has been achieved towards the fulfilment of the intentions and hopes of its founder.

"The fact, also, that the Fund is able this year for the first time to distribute £200,000 among the hospitals and convalescent homes of London is a happy coincidence, and will be regarded by all those who have been impressed by the work of the hospitals, both in war and in peace time, as a landmark in the history of the Fund.

"His Majesty is much gratified by the support which the Fund receives from the public, and, while deeply grateful for the large contributions both in annual subscriptions and gifts to capital from munificent donors, he values very highly the sum derived from those who generously give out of their small incomes, either directly or through the League of Mercy.

"The King notes with satisfaction that the Fund is setting aside grants for improvements which have had to be deferred till after the war, and that the report on Pension Schemes for Hospital Officers, drawn up under the able guidance of Mr. Whittall, is now completed.

"His Majesty knows that the Governors and General Council will join with him in deploring the death of the Chairman of the Convalescent Homes Committee, Dr. Freshfield, who had been connected with the Fund since its foundation.

"His Majesty learns with the utmost regret of the approaching retirement of Sir William Church from the Distribution Committee. The important and onerous work of this Committee has contributed largely to the success of the Fund, and the King feels that we owe a debt of gratitude to Sir William Church for the invaluable services which he has rendered as Chairman of the Committee. His Majesty trusts that he may long remain a member of the General Council.

"It is the earnest hope of the King that the Prince of Wales may be able shortly to take up the Presidency of the Fund, the duties of which, during the minority of the Prince and his absence on service abroad, have been so ably performed by the three Governors.

"His Majesty tenders his sincere thanks to all the members of the Council and Committees, especially to those who have carried out the exacting duties of Chairmen and Secretaries.

"Believe me,
"Yours very truly,
"Stamfordham.

"The Presiding Governor,
"King Edward's Hospital Fund for London."

### The Presiding Governor's Address.

The Fund has come of age since the last distribution meeting of the Council, for it was on February 5, 1897, that his late Majesty King Edward, then Prince of Wales, signed the letter to the public by which it was inaugurated. In that letter £100,000 to £150,000 a year was mentioned as a goal at which to aim. This year the distribution reaches for the first time the sum of £200,000. Of this large sum more than £190,000 has been provided by the current income of the Fund. This includes the receipts from invested funds, which, under the care of the Finance Committee, come in with unfailing regularity even in these difficult times. It also includes legacies, which vary greatly from year to year, and of which the average rather than the receipts for the year is usually taken into account. For the greater part of the balance we are indebted to subscribers and donors, including the numerous contributors to the League of Mercy, the organisers of which deserve a special word of thanks for their splendid efforts throughout the war. The most important individual contributions received during the year have been Mr. Walter Morrison's annual subscription of £5,000, £20,000 received in War Bonds on account of the residue of the estate of the late Brigade Surgeon

### KING EDWARD'S HOSPITAL FUND—

John Law, and £5,254 residue left by the late Mr. N. J. Wilson Blackett. Amongst special contributions to meet the exceptional demands due to the war we have received an additional £800 from Lloyds Bank and £500 from Messrs. John Swire and Son, while Sir John Ellerman has for this year doubled his usual war gift of £500.

But even after taking all these into account we shall probably be obliged, in order to make up the total of £200,000, to draw about £6,000 from the reserve fund created by the legacy of the late Isabella, Countess of Wilton, and the balance of the gift received last year from Viscount Astor.

That the needs of the hospitals justify us in making this effort is shown by the figures in the Fund's Statistical Report. After allowing for payments received towards the treatment of the wounded, the ordinary expenditure falling on the normal sources of hospital revenue in 1917 increased, as compared with 1913, by £311,000. The increase in the grants made by this Fund towards maintenance during the same period has been £45,000.

### Future Needs.

The needs of the hospitals in the immediate future are not likely to diminish. Many beds at present occupied by wounded and partly paid for by the military authorities will be restored to civilian use, and will have to be wholly maintained out of normal sources of income. Renewals and repairs have at many hospitals been necessarily deferred, and will be a cause of heavy expense. Urgent improvements have also been postponed, and extensions are in some cases overdue. The Fund has already set aside several grants for improvements, and more will be required.

### Hospital Extensions.

On the subject of hospital extensions the Distribution Committee include in their report some rather striking figures. Until recently the Fund had discouraged the issue of appeals for post-war building schemes. It was not thought desirable that money should be collected during the war for objects which were not of immediate urgency, and which it might never be possible to carry out. Latterly, however, the Fund has modified its policy by approving the issue of appeals, provided that the terms of the appeal were such that the money collected could be applied to endowment if it could not all be prudently expended on building. The Committee point out in their Report that, if all the schemes which have come to their notice were to be carried out, the number of beds in London hospitals would be increased, as compared with 1913, by at least 1,750. This is a large number, and there may yet be more, since the enlargement of hospitals is often the best form of war memorial, and new ideas of public health are continually increasing the demands on their accommodation.

The Distribution Committee are therefore inviting all the hospitals concerned to let the Fund know how their schemes stand at present, and what their views are as to the future. It is one of the advantages of a Central Fund like this that it can form a sort of clearing-house for ideas in such matters, and can use its influence to co-ordinate the separate efforts of the hospitals.

### Co-ordination of Schemes.

There is, for instance, the question of finance, both for building and for maintenance. Each hospital that contemplates an extension may make a perfectly satisfactory estimate of its own future resources. But the combined total for all the hospitals may be so great as to endanger the success of each separate scheme. There is also the question of geographical distribution. To rearrange the present hospital accommodation would be a gigantic task. But the extensions of existing hospitals can be so distributed as to reduce, as far as possible, the worst inequalities. This, no doubt, is aimed at by each separate scheme, but without a comprehensive knowledge of the general needs, and of all the remedies proposed, the different schemes may sometimes overlap. In these and other ways the friendly co-operation of King Edward's Hospital Fund may be of the greatest assistance.

### Special Grant to a new Hospital.

A remedy for one particular instance of faulty hospital distribution is mentioned in the Distribution Committee's Report. Nine years ago the Committee suggested that a representative movement should be started in Woolwich to provide a general hospital of fair size, and the Council agreed to make an exception to its general rule against making grants to new hospitals. This year we have pleasure in setting aside the sum of £5,000 as a first contribution towards the Woolwich and District War Memorial Hospital Building Scheme.

### Pensions for Hospital Officers.

The Report on Pensions for Hospital Officers, on which a Sub-Committee, under the chairmanship of the well-known Actuary, Mr. W. J. H. Whittall, has been engaged for some time, is on the point of being published by the Executive Committee. The inquiry was undertaken at the request of the Hospital Officers' Association. The Sub-Committee agree that the officers' case is, broadly speaking, made out, and that a satisfactory remedy can only be found in a general scheme. In order to ascertain what the fundamental features of a general scheme should be, they make a survey of various methods of providing pensions already in operation in other services, and the information thus collected and commented on by the Sub-Committee should prove of the greatest interest and assistance to all who are concerned with pension schemes. In the light of the past experience of pension schemes they submit recommendations, though not in all important points unanimously, as to the general lines of a pension scheme for hospital officers and as to the best method of securing the desired end.

### Record Distribution to Convalescent Homes.

This is the first year in which the Convalescent Homes Committee have distributed as much as £10,000, and the number of beds which the Committee secure for the use of patients in London hospitals is now sixty-four, whereas in 1913 it was forty-four.

The Fund has lost one of its oldest members and helpers by the death of Dr. Freshfield, Chairman of the Convalescent Homes Committee, and head of the firm which has acted as honorary solicitors to the Fund since its foundation.

### Sir William Church's Retirement.

The Council will be sorry to hear that this is the last occasion on which the report of the Distribution Committee will be presented by Sir William Church, whose resignation from the Committee has been received and accepted with regret by the Governors. Sir William has been Chairman of the Committee since 1903, during which time that part of the annual distribution which is given to hospitals has increased from £99,000 to £190,000, and the work of the Committee has grown in several ways.

**KING EDWARD'S HOSPITAL FUND—(*continued*).**

We hope Sir William will long remain a member of the General Council, and thus continue to the Fund the support of his name and his experience. The Governors intend to invite Sir John Tweedy to succeed him as Chairman of the Distribution Committee.

I have now to express the thanks of the Governors to the members of the Council and members of Committees, as well as to the visitors and the honorary officers of the Fund, for their work during the past year.

I now beg to move the adoption of the Reports.

The Lord Mayor (Sir Horace Marshall) having seconded the motion, the adoption of the Reports was then put and carried unanimously.

On the motion of the Chief Rabbi (the Rev. J. H. Hertz, D.D.), seconded by Sir Henry Burdett, it was resolved "that the cheques for the grants should be posted on Friday, December 20, except in the case of grants for the reservation of beds in consumption sanatoria during 1919, which should be posted on January 1."

In the absence of the Earl of Bessborough (Chairman of the Executive Committee), Lord Somerleyton (Hon. Secretary) moved the adoption of a further interim Report of the Committee on the subject of rules and regulations.

The motion was seconded by Sir T. Vezey Strong, and carried unanimously.

Arrangements for 1919.

The Marquis of Cambridge then said :—

My Lords and Gentlemen,—I have now to ask one of the Honorary Secretaries to read the list of members of the General Council, members of Committees, and officers whom the Governors intend to appoint for next year. After this it will be necessary to approve the delegation resolutions, which will be laid before the formal meeting in the New Year. In the list of members the Council will miss two familiar names. Mr. Walter Morrison was unable to accept nomination to the Council for 1918, and Mr. Whittall is prevented by ill-health from continuing his services on the Executive Committee, where his work on the Pensions Report has been so arduous and so valuable.

The list having been read,

There was moved by Lord Stamfordham, seconded by Sir John Craggs, and approved, a resolution delegating powers to the Finance Committee, Distribution Committee, and Convalescent Homes Committee.

On the motion of the Chairman of the London County Council (Mr. R. C. Norman), seconded by the President of the Royal College of Physicians (Dr. Norman Moore), a resolution was approved delegating further powers to the Finance Committee.

On the motion of the President of the Royal College of Surgeons (Sir George Makins), seconded by Lord Farquhar, a resolution was approved delegating powers to the Executive Committee.

The Bishop of London, in proposing a vote of thanks to the Marquis of Cambridge for presiding, said his Lordship had always taken the deepest interest in hospitals and the working of King Edward's Fund.

The resolution was seconded by the Rev. Dr. A. E. Garvie, and carried unanimously.

The Marquis of Cambridge having briefly acknowledged the vote of thanks, the proceedings terminated.

